# Molecular epidemiology of carbapenem-resistant hypervirulent *Klebsiella pneumoniae*: risk factors and resistance mechanism of ceftazidime/avibactam in China

**DOI:** 10.3389/fcimb.2025.1698033

**Published:** 2025-12-19

**Authors:** Na Wang, Lexiu Deng, Huiying Li, Na Jia, Xiaocui Peng, Jianliang Chang, Jiatong Hao, Jianhua Tang, Chunmei Lei, Bu Wang, Jianhua Liu, Wei Zhang

**Affiliations:** 1 Microbiology Department, The First Affiliated Hospital of Hebei North University, Hebei North University, Zhangjiakou, Hebei, China; 2 Infection Management Department, The First Affiliated Hospital of Hebei North University, Hebei North University, Zhangjiakou, Hebei, China; 3 Respiratory Department, The First Affiliated Hospital of Hebei North University, Hebei North University, Zhangjiakou, Hebei, China; 4 Obstetrics and Gynecology Department, The First Affiliated Hospital of Hebei North University, Hebei North University, Zhangjiakou, Hebei, China; 5 Clinical Laboratory, Tianjin Nankai Tianyun Hospital, Tianjin, China; 6 Department of Pharmacy, The First Affiliated Hospital of Hebei North University, Hebei North University, Zhangjiakou, Hebei, China; 7 Central Laboratory, The First Affiliated Hospital of Hebei North University, Hebei North University, Zhangjiakou, Hebei, China

**Keywords:** carbapenem-resistant hypervirulent *Klebsiella pneumoniae*, ceftazidime/avibactam resistance, ST11 clone, molecular epidemiology, China

## Abstract

**Background:**

Carbapenem-resistant hypervirulent *Klebsiella pneumoniae* (CR-hvKP) represents a critical public health threat in China, characterized by the convergence of multidrug resistance and hypervirulence. The emergence of ceftazidime/avibactam (CZA) resistance further complicates clinical management. This study aimed to elucidate the molecular epidemiology, risk factors, and resistance mechanisms of CZA resistance in CR-hvKP across China, providing evidence for targeted interventions.

**Methods:**

A single-center molecular epidemiological analysis was conducted on 81 Carbapenem-resistant *Klebsiella pneumoniae* (CRKP) clinical isolates collected. All isolates underwent whole-genome sequencing for MultiLocus Sequence Typing, capsule typing, and identification of resistance genes (*bla*KPC-2 and *blaNDM-1*) and virulence factors (*iucA*, *iroB*, *rmpA*, *rmpA*2, and *peg-*344). CZA resistance mechanisms were investigated through broth microdilution minimum inhibitory concentration (MIC) testing and bioinformatics analysis. *Galleria mellonella* infection models were employed to assess virulence potential. Risk factors were analyzed using multivariate regression of clinical variables. Phylogenetic reconstruction employed single-nucleotide polymorphism-based analysis.

**Results:**

ST11 accounted for 96.15% (50/52) of CR-hvKP isolates, with K64 being the predominant capsule type (92.31%, 48/52). Additionally, 98.77% (80/81) of CRKP carried ≥1 virulence gene; 64.2% (52/81) of isolates with all five virulence genes exhibited lethality. *Galleria mellonella* revealed that the survival rate of CR-hvKP was lower than that of carbapenem-resistant non-hypervirulent *Klebsiella pneumoniae* (p<0.05). Antibiotic usage time (odds ratio [OR]=1.076, 95% confidence interval [CI]: 1.026–1.138), carbapenem antibiotic (OR = 0.117, 95% CI: 0.02266–0.4602), and malignant tumors (OR = 65.1, 95% CI: 7.078–1798) predicted CR-hvKP infection. Transferable *bla*KPC-2 on IncFII/IncR plasmids conferred CZA resistance (MIC>128 mg/L) without compromising carbapenem resistance, facilitated by a unique genetic context (TnpR_Tn3-ISKpn27-*bla*KPC-2-ISKpn6).

**Conclusion:**

China faces a rapid dissemination of ST11 CR-hvKP clones carrying diversified CZA resistance mechanisms. The convergence of hypervirulence and resistance in ST11 lineages—accelerated by invasive procedures and international transmission—demands enhanced genomic surveillance. CZA resistance arises through multiple pathways, necessitating combination therapies and stewardship programs limiting prolonged CZA use. Our findings underscore an urgent need for rapid diagnostics targeting emergent resistance determinants and infection control measures to contain high-risk clones.

## Graphical Abstract

**Figure:**
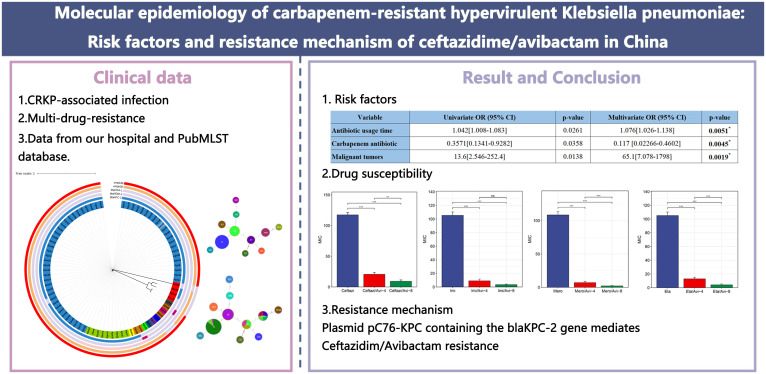

## 1 Introduction

Carbapenem-resistant *Klebsiella pneumoniae* (CRKP) has become a major global public health threat, and the prevalence of carbapenem-resistant hypervirulent *Klebsiella pneumoniae* (CR-hvKP) has sharply increased in China, leading to high morbidity and mortality rates due to the lack of effective treatment options (Zhang et al., 2018; Yang et al., 2022). *Klebsiella pneumoniae* Carbapenemase (KPC) belongs to class A serine carbapenemases and is the main mechanism of resistance in Enterobacteriaceae. The proportion of *bla*KPC-2 producing CRKP in China exceeds 70% (Yang et al., 2022). In China, ST11 CRKP has become the major clonal type (Hu et al., 2024), whereas in the United States and Europe, ST258/512 is predominant (Tryfinopoulou et al., 2023). Owing to ceftazidime/avibactam (CZA) having good *in vitro* sensitivity and safety against serine carbapenemases, it has become the last line of defense against CRKP infections. However, CRKP can generate *bla*KPC mutations via various mechanisms, causing resistance to CZA (Pu et al., 2023a; Shi et al., 2024). Research has found that the diversity of the CRKP genome is mainly due to horizontal transfer, including plasmids, phages, integration, and binding elements. The spread of *bla*KPC-2 is typically mediated by two mobile elements, Tn4401 and NTEKPC (Yang et al., 2021).

Globally, CR-hvKP is increasingly reported, and most clinical cases occur in Asia, particularly in China, where studies have shown that the prevalence of CR-hvKP has increased from 28.2% in 2016 to 45.7% in 2020 (Hu et al., 2024), causing serious nosocomial infection in the intensive care unit ward (Gu et al., 2018). CR-hvKP virulence factors include capsules, siderophores, virulence plasmids, and other virulence genes, which help differentiate hypervirulent strains (Pu et al., 2023b). Various complex mechanisms have led to the rapid spread of strains in the clinic (Pu et al., 2023b). Previous studies have mainly focused on resistant or virulent strains of hvKP, with little attention paid to the correlations among virulence genes, resistance genes, and antimicrobial susceptibility of CR-hvKP. Therefore, this study aimed to summarize the molecular epidemiological characteristics of CR-hvKP isolated from this region and discuss the evolution of virulence and resistance genes in CR-hvKP and their relationship with clinical phenotypes.

## 2 Materials and methods

### 2.1 Isolate and antimicrobial susceptibility testing

**Figure 1:**
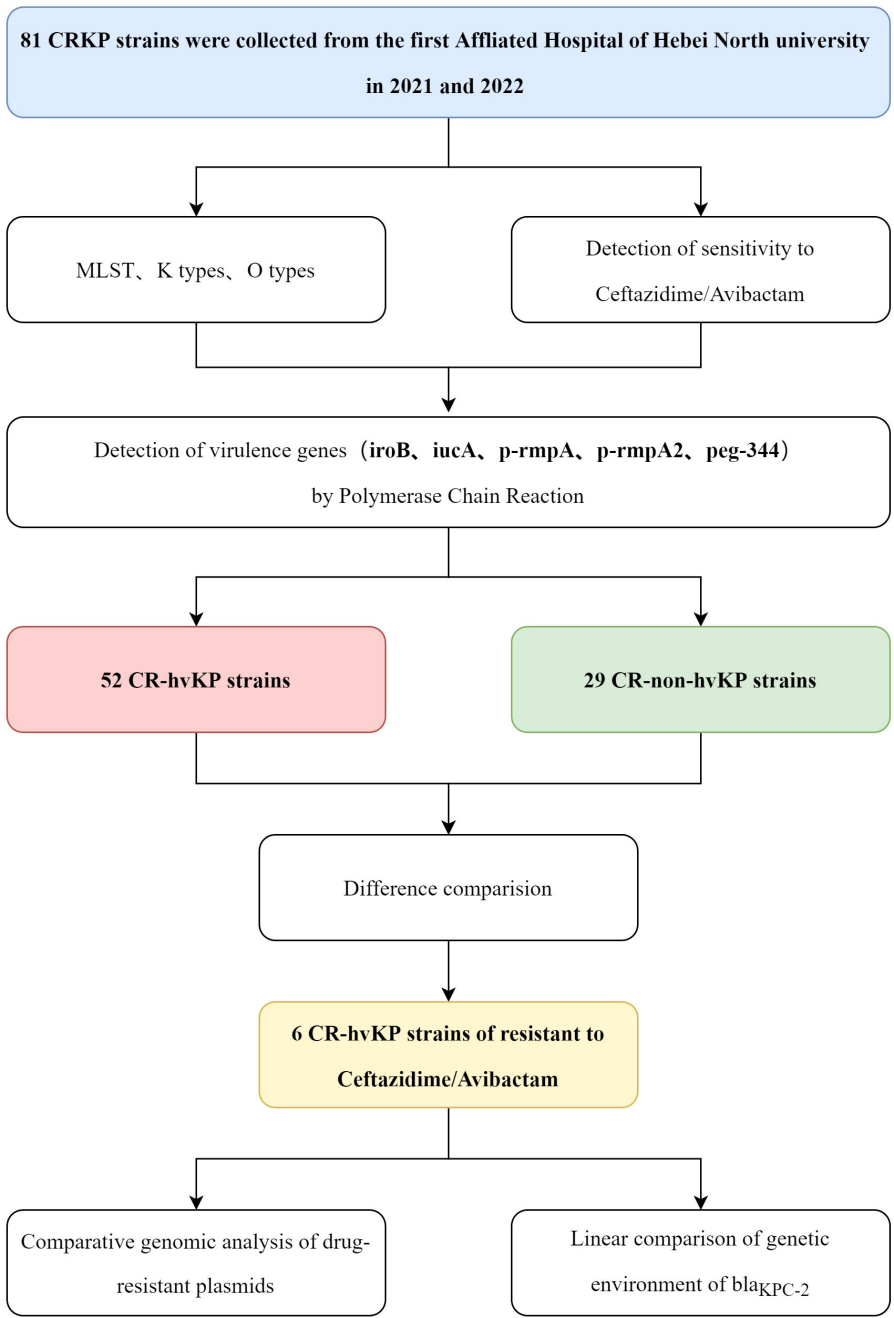
Design and experimental flowchart of this study.

In this study, 81 non-duplicate CRKP strains were collected from the First Affiliated Hospital of Hebei North University between 2021 and 2022 for analysis (**Figure 1**). The PubMLST database was downloaded for comparative analysis of *Klebsiella pneumoniae* containing *bla*KPC-2 and/or *bla*NDM-1 in November 2024 (https://bigsdb.pasteur.fr/klebsiella/). This study employed the microbroth dilution method recommended by the Clinical and Laboratory Standards Institute to perform *in vitro* AST of 81 CRKP strains. Sensitivity, intermediate resistance, and resistance were determined according to the Clinical and Laboratory Standards Institute-M100 ED33 guidelines. A minimum inhibitory concentration (MIC) of ≥4 mg/L for imipenem or meropenem against *KP* was defined as CRKP.

### 2.2 Polymerase chain reaction

Polymerase chain reaction experiments were conducted on the 81 collected CRKP strains using the following reaction system: The total reaction volume was 25 μL, which included 12.5, 8.5, 1, 1, and 2 μL of 2x Taq Plus Master Mix II (Dye Plus), deionized water, forward primer, reverse primer, and template DNA, respectively. The reaction program was set as follows: initial denaturation at 95°C for 3 min, denaturation at 95°C for 15 s, annealing at 60°C for 20 s, and extension at 72°C for 60 s, with a total of 30 cycles. After the reaction, agarose gel electrophoresis was performed to record the experimental results.

### 2.3 Whole genome sequencing and annotation

Genomic DNA was extracted using the sodium dodecyl sulfate method, and the harvested DNA was analyzed through agarose gel electrophoresis. Quantitative analysis of the DNA was performed using a Qubit^®^ 2.0 fluorometer (Thermo Scientific). Sequencing libraries were prepared using the NEBNext Ultra DNA Library Prep Kit (Illumina, USA), and index codes were added to classify the sequences for each sample. Whole-genome sequencing was performed on an Illumina NovaSeq PE150 platform using a 2 × 150 bp paired-end strategy.

The genome was annotated using Prokka V1.14.6, and resistance, virulence genes, and plasmid replicons were predicted using Abricate V1.0.1. Capsular serotypes were predicted using PathogenWatch (https://pathogen.watch). Multilocus sequence typing (MLST) of all strains was conducted using the Pasteur database (https://bigsdb.pasteur.fr/klebsiella/), and newly identified sequence typing (ST) was submitted to the MLST database administrator for approval and were assigned ST numbers. A minimum spanning tree based on the allelic difference between isolates of the seven housekeeping genes was constructed using PHYLOViZ (Ribeiro-Gonçalves et al., 2016).

### 2.4 Phylogenetic analysis

Single-nucleotide polymorphisms were extracted using Snippy v4.6.0 (https://github.com/tseemann/snippy) to generate a core genome alignment. This core genome alignment was employed to construct a maximum likelihood phylogenetic tree using FastTree V2.1.11-2, with *KP* subsp. *pneumoniae* HS11286 (GCA_000240185.2) as the reference genome. The resulting phylogenetic tree was visualized using iTOL (https://itol.embl.de/) (Letunic and Bork, 2024).

### 2.5 *Galleria mellonella* killing assay

Bacterial suspensions were adjusted to a 0.5 McFarland standard (approximately 1.5 × 10^8^ CFU/mL) and then serially diluted in phosphate-buffered saline (PBS) to obtain target concentrations. A 10 µL volume of each dilution was injected into the hemocoel of Galleria mellonella larvae via the last right proleg, resulting in final inocula of approximately 1.5 × 10^5^ and 1.5 × 10^4^ CFU/larva (corresponding to 10-fold and 100-fold dilutions of the standard suspension, respectively). The control group received an equal volume of sterile PBS. After injection, larvae were incubated at 37°C, and survival was monitored every 2 hours for 48 hours. The mortality rate was calculated at the endpoint of the observation period.

### 2.6 Antibiotic-resistant plasmids and mobile genetic elements containing the *bla*KPC-2 gene

The assembled contigs of six CR-hvKP strains resistant to cefotaxime/avibactam were input into the VRprofile2 pipeline (https://tool2-mml.sjtu.edu.cn/VRprofile/home.php) to divide the contigs into chromosome or plasmid fragments and identify whether they carry resistance genes. To further identify plasmids carrying resistance genes, we compared these contigs with the NCBI database (https://www.ncbi.nlm.nih.gov/) using BLASTn to search for reference sequences. For the plasmid resistant to cefotaxime/avibactam, we used pC76 KPC (NZ_CP080299.1) with a coverage of 93.82% and an identity of 99.37% as a reference sequence to study the structure of the plasmid. MAUVE (Darling et al., 2004) was used to identify all contigs located in drug-resistant plasmids by comparing the contigs of the strain with the reference plasmid pC76 KPC (NZ_CP080299.1). Plasmid maps were presented using BRIG (Alikhan et al., 2011). To determine whether contigs carrying the *bla*KPC-2 gene all contain insertion and repeat sequences, we submitted them to Isfinder (Siguier et al., 2006) (https://www-is.biotoul.fr/index.php). Easyfig (Sullivan et al., 2011) was used to visualize the structure of the *bla*KPC-2 gene.

### 2.7 Statistical methods

Categorical variables were compared between the CR-hvKP and carbapenem-resistant non-hypervirulent *Klebsiella pneumoniae* (CR-non-hvKP) groups using the Chi-square test, and presented as numbers (percentages). The Log-rank (Mantel-Cox) test was used to analyze survival differences in the *Galleria mellonella* infection model. Differences in AST results among groups were analyzed using the Wilcoxon rank-sum and Kruskal–Wallis tests, as appropriate. Spearman’s correlation analysis was conducted to explore the relationships between plasmids and virulence genes, plasmids and resistance genes, resistance genes and clinical data, and virulence genes and clinical data.

Univariate logistic regression analyses were performed using IBM SPSS Statistics for Windows, version 27.0 (IBM Corp., Armonk, N.Y., USA) to identify potential risk factors for CR-hvKP infection. Variables with a p-value < 0.05 in the univariate analysis were included in the multivariate model. The multivariate logistic regression analysis was conducted using GraphPad Prism (version 8). The goodness-of-fit of the final multivariate model was assessed using the Hosmer–Lemeshow test, and its discriminatory power was evaluated through the area under the receiver operating characteristic curve. A two-tailed p-value < 0.05 was considered statistically significant.

Statistical analyses were performed using IBM SPSS Statistics for Windows, version 27.0 (IBM Corp., Armonk, N.Y., USA) and GraphPad Prism 8 (GraphPad Software, LaJolla, CA, USA). Visualization was performed using Xiantao Academic (https://www.xiantaozi.com/), the Wekemo Bioincloud (https://www.bioincloud.tech) (Gao et al., 2024), and Origin 2021 (OriginLab Co., MA, USA). Plots were generated using R software (v.4.2.2) with the ggplot2 (v.3.3.6) (Villanueva and Chen, 2019) package through Hiplot Pro (https://hiplot.com.cn/).

### 2.8 Ethical statements

The study was approved by the Ethics Committee of the First Affiliated Hospital of Hebei North University (ethical approval No. K2019147), which waived the requirement of written informed consent from patients. All strains are part of the routine laboratory procedures of the hospital and do not involve any human genetic resources. This study was conducted in accordance with the principles outlined in the Declaration of Helsinki.

## 3 Results

### 3.1 Molecular epidemiology

**Figure 2:**
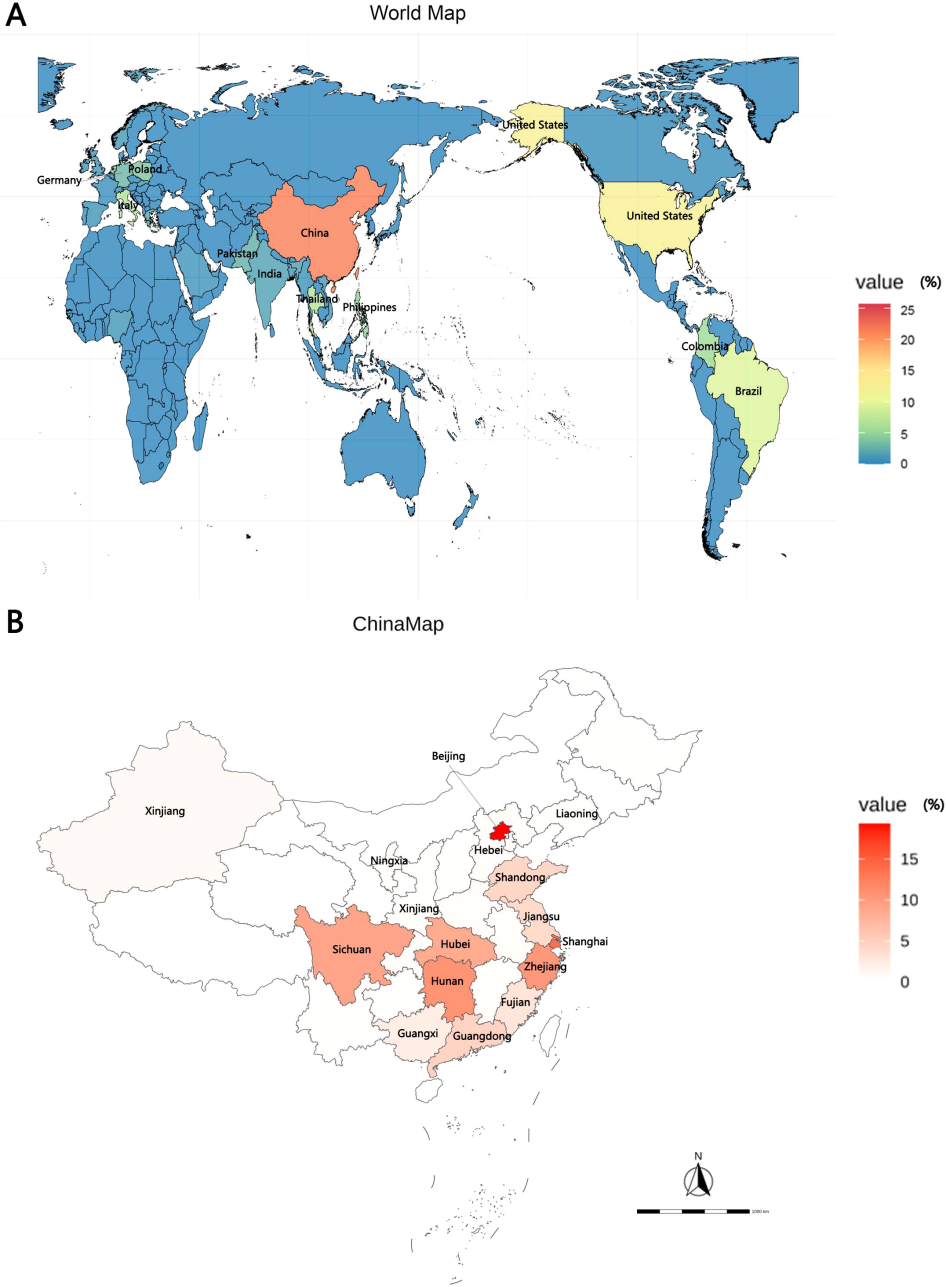
Global and Chinese distribution of CRKP infection by country. **(A)** Global distribution of CRKP infection. **(B)** Chinese distribution of CRKP infection. CRKP, carbapenem-resistant *Klebsiella pneumonia*.

**Figure 3:**
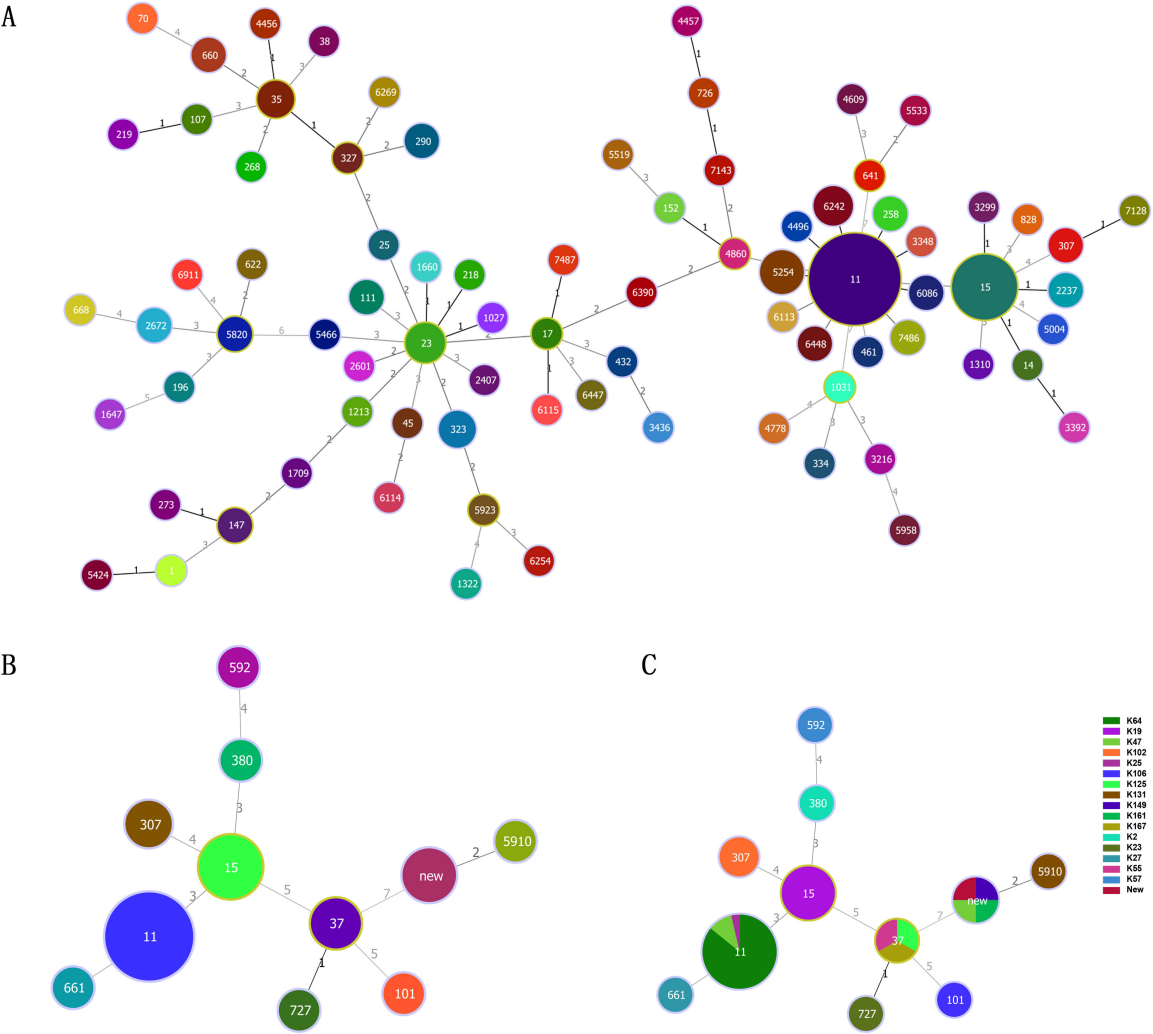
ST-type minimum-spanning tree. **(A)** Genetic relationship of carbapenem-resistant *Klebsiella pneumoniae* isolates of China. **(B)** Genetic relationship of carbapenem-resistant *Klebsiella pneumonia* isolates of this study; **(C)** Genetic relationship between MLST and K-type of carbapenem-resistant *Klebsiella pneumonia* isolates of this study. The size of the dots is proportional to the number of strains. Numbers on the connecting line represent genetic distance. MLST, MultiLocus Sequence Typing.

**Figure 4:**
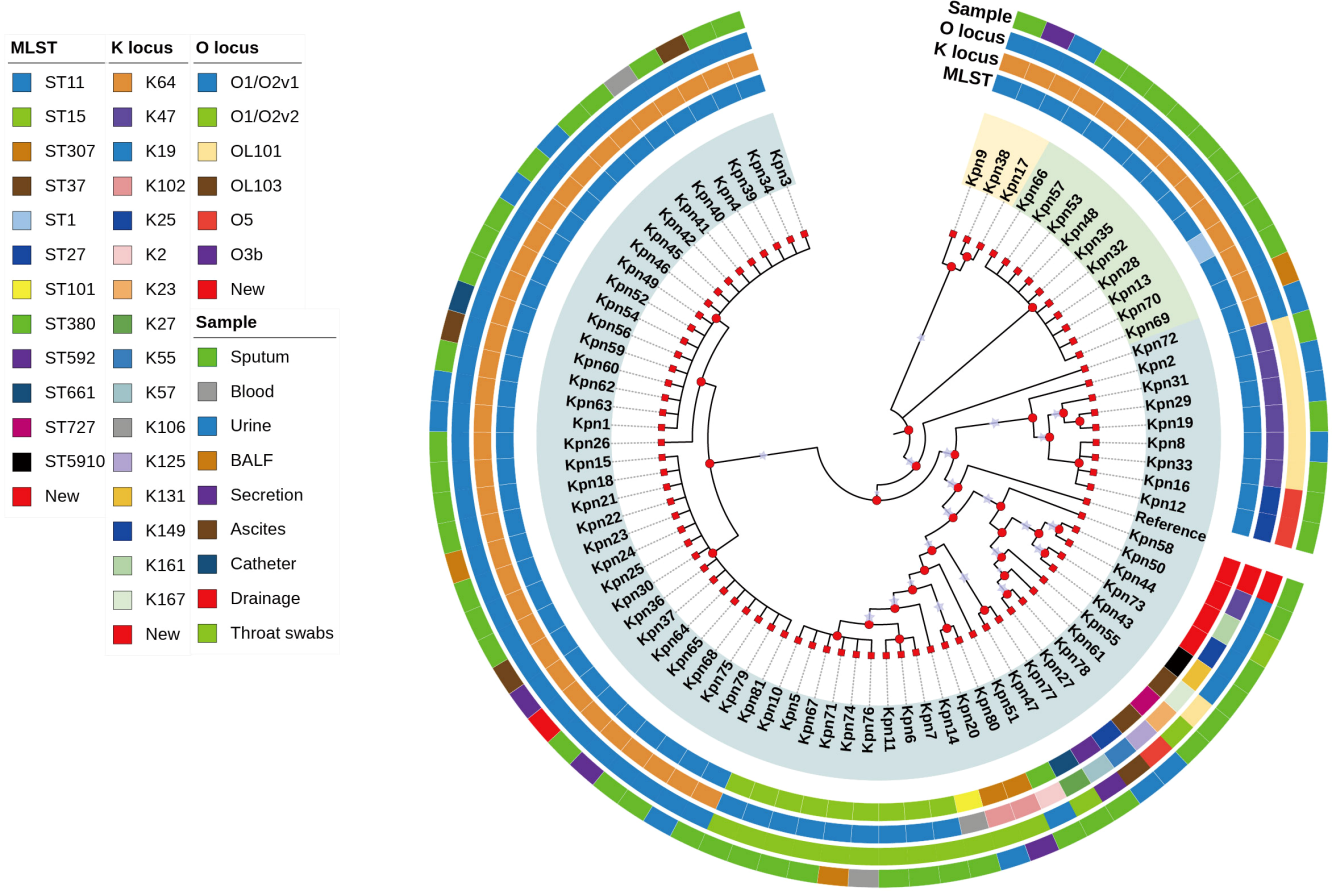
Evolutionary relationship diagram of 81 CRKP strains. CRKP: carbapenem-resistant *Klebsiella pneumonia*.

**Figure 5:**
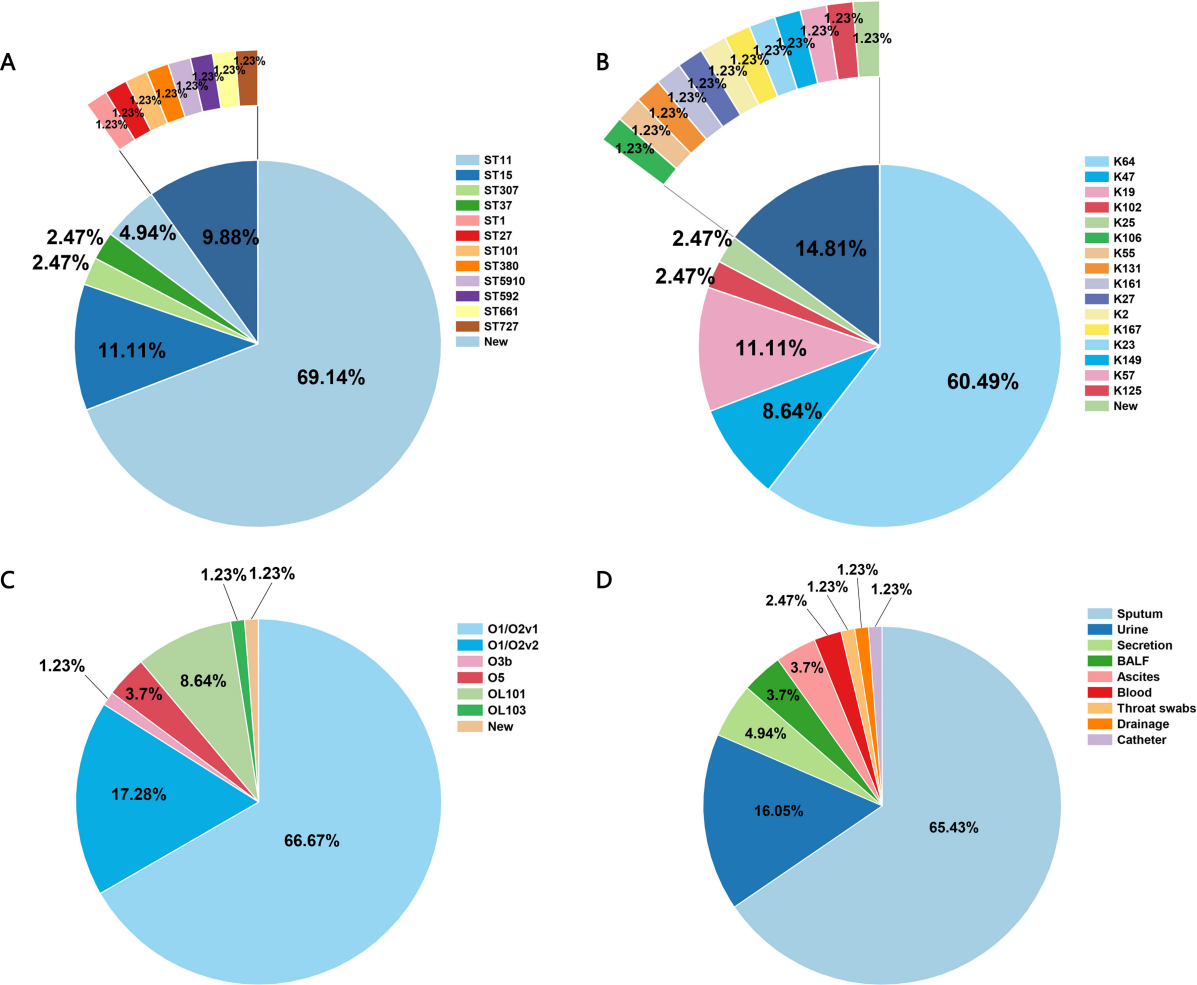
Overall distribution of 81 CRKP strains. **(A)** ST distribution; **(B)** K-type distribution; **(C)** O-type distribution; **(D)** Sample distribution CRKP: carbapenem-resistant *Klebsiella pneumonia;* ST, sequence typing.

The global prevalence of CRKP is highlighted in **Figure 2A**, with China, the United States, and Brazil being the most affected countries. Within China, the highest frequency of CRKP isolates was reported in Beijing, followed by Shanghai and Hunan (**Figure 2B**). The molecular epidemiology at our hospital reflected this national trend (**Figure 3A**), with ST11 being the predominant type (69.14%), followed by ST15 (11.11%) among the 12 STs detected (**Figure 3B**). Capsular (K) antigen serotyping revealed 16 different types, with K64 being the most common (60.49%, 49/81). Similarly, lipopolysaccharide (O) antigen serotyping identified O1/O2v1 as the predominant serotype (66.67%, 54/81). These strains were primarily isolated from sputum (65.43%, 53/81), followed by urine (16.05%, 13/81) and secretions (4.94%, 4/81) (**Figure 3C**), which is consistent with provincial surveillance data (Wang et al., 2023, 2022). Phylogenetic analysis showed that evolutionary clustering was associated with ST type rather than specimen source. The primary clusters identified were ST11-K64-O1/O2v1, ST11-K47-OL101, and ST15-K19-O1/O2v2. Notably, ST11 strains diverged into three distinct branches based on serotype (ST11-K64-O1/O2v1, ST11-K47-OL101, and ST11-K25-other), suggesting divergent evolution within this lineage (**Figure 4**). The overall distributions of ST, K-type, O-type, and sample sources among the 81 CRKP strains are summarized in **Figure 5**.

### 3.2 Multivariate logistic regression analysis of independent risk factors for CR-hvKP infection

For the convenience of analysis, we categorized 81 CRKP strains into the following two groups: the high virulence *(rmpA, rmpA*2*, iucA, iroB*, and *peg-*344) (Russo et al., 2018) and low virulence groups. We collected clinical data from 81 patients with CRKP, including patient Basic Data, Department, Underlying diseases, Infection type, Invasive procedures and devices, Antimicrobial exposure, and Outcomes. After analysis, significant differences (p<0.05) were found between the two groups of patients regarding Antimicrobial usage time, Pulmonary disease, Malignant tumors, and Carbapenem antimicrobial exposure and Outcomes. Logistic multiple regression analysis was conducted on the items with significant differences mentioned above, and it was found that Antibiotic usage time, Carbapenem antibiotic exposure, and Malignant tumors were independent risk factors for CR-hvKP infection (**Tables 1**, **2**).

**Table 1:** Clinical characteristics of CR-hvKP and CR-non-hvKP.

Factors	CR-hvKP(n=52)	CR-non-hvKP(n=29)	χ²/t	P-value
Basic Data				
Male	38(73.1%)	20 (69.0%)	0.155	0.694
female	14(26.9%)	9 (31.0%)	0.155	0.694
Age^a^	66.62 ± 15.075	67.52 ± 13.230	0.269	0.788
WBC(*10^9^)^a^	11.099 ± 5.654	11.079 ± 3.418	-0.017	0.987
NEU(*10^9^)^a^	79.551 ± 16.787	80.683 ± 14.777	0.304	0.762
Admission to ICU^a^, days	16.94 ± 19.467	18.62 ± 31.409	0.297	0.707
Length of stay in hospital^a^, days	30.90 ± 18.922	31.21 ± 28.099	0.058	0.954
Antibiotic usage time^a^, days	28.00 ± 18.779	19.10 ± 11.821	-2.306	0.024^*^
Admission temperature^a^(°C)	36.98 ± 1.051	36.62 ± 0.547	-1.718	0.090
Department				
ICU	38 (72.08%)	18 (62.07%)	1.057	0.304
internal medicine	11 (21.15%)	10 (34.48%)	1.722	0.189
surgical department	3 (5.77%)	1 (3.45%)	0.214	0.644
Underlying diseases				
Diabetes	9 (17.3%)	5 (17.2%)	0.000	0.994
Hypertension	26 (50.0%)	13 (44.8%)	0.200	0.655
Cardiovascular disease	20 (38.5%)	8 (27.5%)	0.974	0.324
Pulmonary disease	17 (32.7%)	2 (6.9%)	6.900	0.009^*^
Malignant tumors	14 (26.9%)	0 (0)	9.439	0.002^*^
Infection type				
Pneumonia	34 (65.4%)	23 (79.3%)	1.732	0.188
Urinary infection	9 (17.3%)	4 (13.8%)	0.009	0.922
Bacteremia	1 (1.9%)	1 (3.4%)	0.000	1.000
Other	8 (15.4%)	1 (3.4%)	1.613	0.204
Invasive procedures and devices				
Tracheal intubation	26 (50.0%)	13 (44.8%)	0.200	0.655
Central intravenous catheter	18 (34.6%)	9 (31.0%)	0.107	0.734
Antibiotic exposure				
Cephalosporins	27 (51.9%)	16 (55.2%)	0.079	0.779
Carbapenem antibiotic	24 (46.2%)	3 (10.3%)	10.743	0.001^*^
β-lactam/β-lactamase inhibitors	21 (40.1%)	8 (27.6%)	1.327	0.249
Fluoroquinolones	18 (34.6%)	8 (27.6%)	0.422	0.516
Aminoglycosides	4 (7.7%)	4 (13.8%)	0.244	0.621
Glycopeptides	6 (11.5%)	3 (10.3%)	0.027	0.870
Hormone	11 (21.2%)	4 (13.8%)	0.668	0.414
Outcomes				
Positive outcome	33 (63.5%)	25 (86.2%)	4.737	0.030^*^
Negative outcome	19 (36.5%)	4 (13.8%)	4.737	0.030^*^

**^a^**Age, WBC, NEU, Admission to ICU, Length of stay in hospital, Antibiotic usage time and Admission temperature as mean and standard deviation (SD), ^*^Bold font means p < 0.05; CR-hvKP, carbapenem-resistant hypervirulent *Klebsiella pneumoniae*; CR-nom-hvKP, carbapenem-resistant non-hypervirulent *Klebsiella pneumoniae*.

**Table 2:** Multivariate logistic regression analysis of independent risk factors for CR-hvKP infection.

Variable	Univariate OR (95% CI)	p-value	Multivariate OR (95% CI)	p-value
Antibiotic usage time	1.042 (1.008–1.083)	0.026	1.076 (1.026–1.138)	0.005^*^
Carbapenem antibiotic	0.3571 (0.1341–0.9282)	0.036	0.117 (0.02266–0.4602)	0.005^*^
Pneumonia disease	0.2975 (0.0995–0.8518)	0.025	1.594 (0.8704–3.336)	0.159
Malignant tumors	13.6 (2.546–252.4)	0.014	65.1 (7.078–1798)	0.002^*^
Outcomes	3.167 (1.339–8.694)	0.014	0.2459 (0.05379–0.9333)	0.050

The overall performance of the multivariate logistic regression model was good. The model demonstrated a good fit to the data, as indicated by a non-significant Hosmer–Lemeshow test result (χ² = 3.250, p = 0.918). Furthermore, the model exhibited excellent discriminatory power, with an area under the receiver operating characteristic (ROC) curve of 0.808 (95% CI: 0.714–0.901; p < 0.001).

^*^Bold font means p < 0.05; OR, Odds Ratio; CI, confidence interval; CR-hvKP, carbapenem-resistant hypervirulent *Klebsiella pneumonia.*

### 3.3 Antimicrobial susceptibility tests

**Figure 6:**
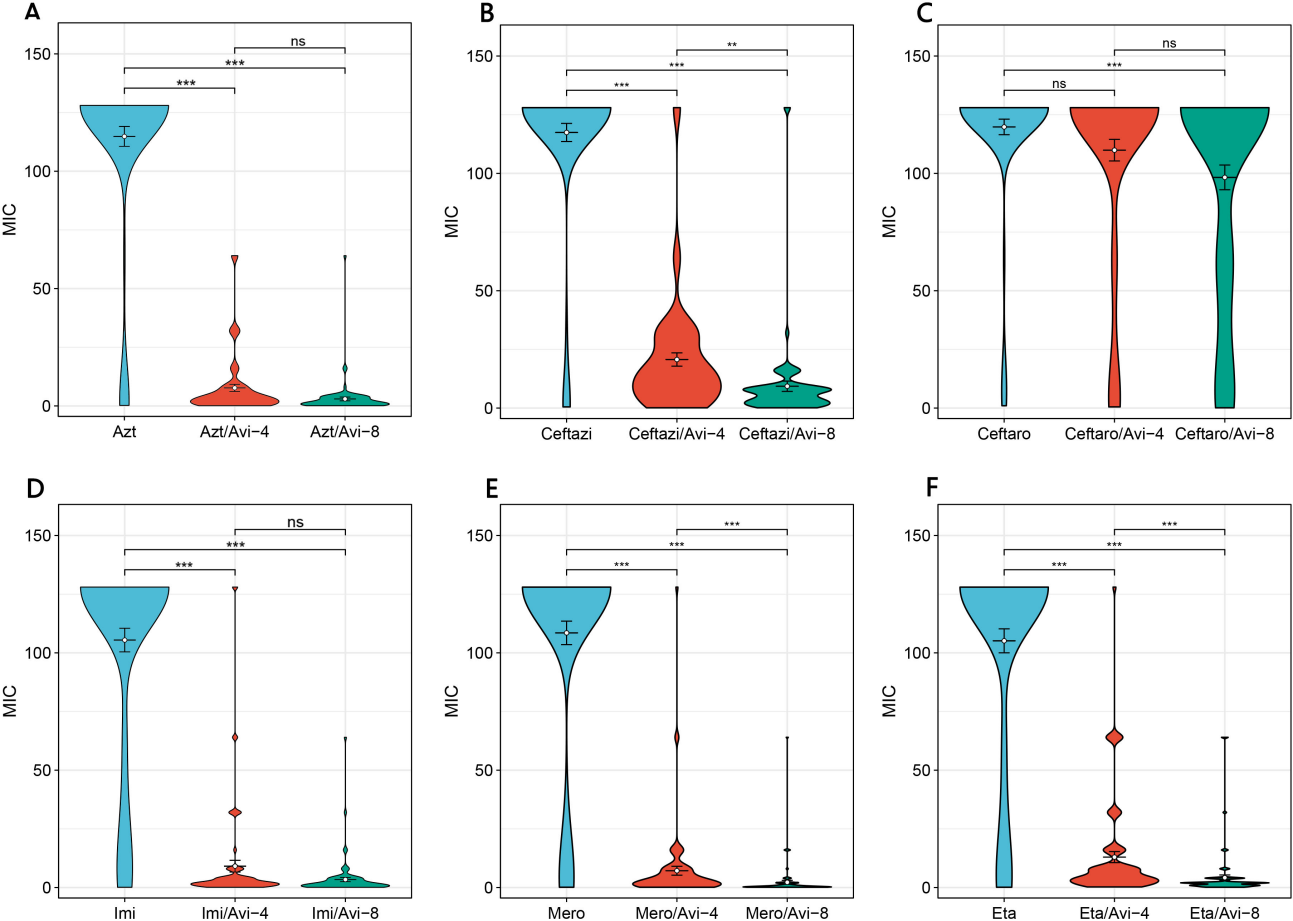
Comparison of MIC values of CRKP strains against novel antibiotics β-lactam/β-lactase inhibitors. **(A)** MIC values of Aztreonam, Aztreonam/Avibactam-4, and Aztreonam/Avibactam-8; **(B)** MIC values of Ceftazidim, CZA-4, and CZA-8; **(C)** MIC values of Ceftaroline, Ceftaroline/Avibactam-4, and Ceftaroline/Avibactam-8; **(D)** MIC values of Imipenem, Imipenem/Avibactam-4, and Imipenem/Avibactam-8; **(E)** MIC values of Meropenem, Meropenem/Avibactam-4, and Meropenem/Avibactam-8; **(F)** MIC values of Ertapenem, Ertapenem/Avibactam-4, and Ertapenem/Avibactam-8. ns represents not significant (p ≥ 0.05), **represents p < 0.01, and ***represents P < 0.001. CRKP: carbapenem-resistant Klebsiella pneumoniae; MIC: minimum inhibitory concentration; ST, sequence typing; Azt, Aztreonam; Ceftazi, Ceftazidime; Ceftaro,Ceftaroline; Imi, Imipenem; Mero, Meropenem; Eta, Ertapenem; Avi, Avibactam; CZA, Ceftazidime-avibactam.

We conducted a new antibiotics combination β-lactam/β-lactase inhibitor, tigecycline and polymyxin susceptibility test on 81 CRKP strains, including Aztreonam/Avibactam, Ceftaroline/Avibactam, CZA, Imipenem/Avibactam, Meropenem/Avibactam, and Ertapenem/Avibactam, and classified them into high (4 μg/mL) and low (8 μg/mL) concentration inhibitor groups. Except for Ceftaroline, significant differences (p<0.05) were found in the drug sensitivity results of the other five antibiotics after adding low-concentration avibactam inhibitors (**Figure 6C**). The MIC values of CZA, Meropenem/Avibactam, and Ertapenem/Avibactam increased as the inhibitor concentration rose (p<0.05) (**Figures 6B, E, F**). However, no significant change was observed in MIC values after adding high-concentration inhibitors, including Aztreonam/Avibactam and Imipenem/Avibactam (**Figures 6A, D**). The resistance rate of polymyxin (79.01%) was lower than that of tigecycline (96.30%), and the resistance rate and MIC range values of the CR-hvKP group were generally higher than those of the CR-non-hvKP group (**Tables 3**, **4**).

**Table 3:** Drug susceptibility characteristics of novel enzyme inhibitor antibiotics.

Antibiotic	R	S	I	MIC	MIC50	MIC90
Aztreonam	91.36%	8.64%	0.00%	>128	>128	>128
Aztreonam /Avibactam-4	13.58%	72.84%	13.58%	4	1	2
Aztreonam /Avibactam-8	3.70%	95.06%	1.23%	1	0.5	0.5
Ceftazidime	92.59%	7.41%	0.00%	>128	>128	>128
CZA-4	58.02%	41.98%	0.00%	16	4	8
CZA-8	14.81%	85.19%	0.00%	8	2	4
Ceftaroline	98.77%	0.00%	1.23%	>128	>128	>128
Ceftaroline/Avibactam-4	96.30%	3.70%	0.00%	>128	>128	>128
Ceftaroline/Avibactam-8	92.59%	4.94%	2.47%	>128	64	64
Imipenem	91.36%	7.41%	1.23%	>128	>128	>128
Imipenem/Avibactam-4	38.27%	30.86%	30.86%	2	0.5	1
Imipenem/Avibactam-8	29.63%	53.09%	17.28%	1	0.5	0.5
Meropenem	88.89%	11.11%	0.00%	>128	>128	>128
Meropenem/Avibactam-4	45.68%	35.80%	18.52%	2	1	1
Meropenem/Avibactam-8	11.11%	75.31%	13.58%	0.5	<0.125	0.25
Ertapenem	91.36%	7.41%	1.23%	>128	>128	>128
Ertapenem/Avibactam-4	85.19%	7.41%	7.41%	4	2	2
Ertapenem/Avibactam-8	56.79%	19.75%	23.46%	2	0.5	1
Tigecycline	96.30%	3.70%	0.00%	4	1	2
Polymyxin	79.01%	20.99%	0.00%	8	2	4

R, drug resistance rate; S, sensitivity rate; I, Intermediary rate; CZA, ceftazidime/avibactam.

MIC, minimum inhibitory concentration; MIC 50, minimum inhibitory concentration required to inhibit the growth of 50% of isolates; MIC 90, minimum inhibitory concentration required to inhibit the growth of 90% of isolates.

**Table 4:** Resistance rates of CR-hvKP and CR-non-hvKP.

Antibiotic	CR-hvKP		CR-non-hvKP	
	R%	MIC range	R%	MIC range
Aztreonam	98.08%	0.25–>128	79.31%	0.25–>128
CZA-4	17.31%	0.125–64	6.90%	<0.125–64
CZA-8	1.92%	<0.125–64	6.90%	<0.125–16
Ceftazidime	98.08%	0.5–>128	82.76%	0.5–>128
Ceftazidime/Avibactam-4	71.15%	1–64	34.48%	<0.125–>128
Ceftazidime/Avibactam-8	13.46%	0.5–16	17.24%	<0.125–>128
Ceftaroline	100.00%	16–>128	96.55%	1–>128
Ceftaroline/Avibactam-4	100.00%	4–>128	89.66%	0.5–>128
Ceftaroline/Avibactam-8	100.00%	4–>128	79.31%	<0.125–>128
Imipenem	96.15%	<0.125–>128	82.76%	0.5–64
Imipenem/Avibactam-4	46.15%	0.25–64	24.14%	<0.125–>128
Imipenem/Avibactam-8	32.69%	<0.125-64	24.14%	<0.125–>128
Meropenem	100.00%	16–>128	68.97%	<0.125–>128
Meropenem/Avibactam-4	53.85%	0.125–16	31.03%	<0.125–>128
Meropenem/Avibactam-8	9.62%	<0.125–16	13.79%	<0.125–>128
Ertapenem	98.08%	0.25–>128	79.31%	<0.125–64
Ertapenem/Avibactam-4	96.15%	0.25–64	65.52%	<0.125–128
Ertapenem/Avibactam-8	69.23%	<0.125–64	34.48%	<0.125–64
Tigecycline	96.15%	0.5–64	96.55%	0.25–128
Polymyxin	86.54%	<0.125–128	65.52%	<0.125–64
Cefepime	88.46%	>16–>32	100.00%	<1–>32
Cefotaxime	80.77%	>2–>32	66.00%	<0.5–>32
Amoxicillin/clavulanate	88.46%	>16/8	100.00%	<4/2–>16/8
Ampicillin/Sulbactam	96.15%	>16/8	100.00%	8/4–>16/8
Levofloxacin	95.83%	>4–>8	100.00%	<1–>8
Ciprofloxacin	92.31%	>2–>16	100.00%	<1–>16
Amikacin	23.08%	<8–>32	92.00%	<8–>32
Gentamicin	73.08%	<2–>8	90.00%	<1–>8

R, drug resistance rate; CR-hvKP, carbapenem-resistant hypervirulent *Klebsiella pneumoniae*; CR-nom-hvKP, carbapenem-resistant non-hypervirulent *Klebsiella pneumoniae*; MIC, minimum inhibitory concentration; CZA, ceftazidime/avibactam.

### 3.4 Antimicrobial resistance genes, virulence genes, and plasmids of CR-hvKP

**Figure 7:**
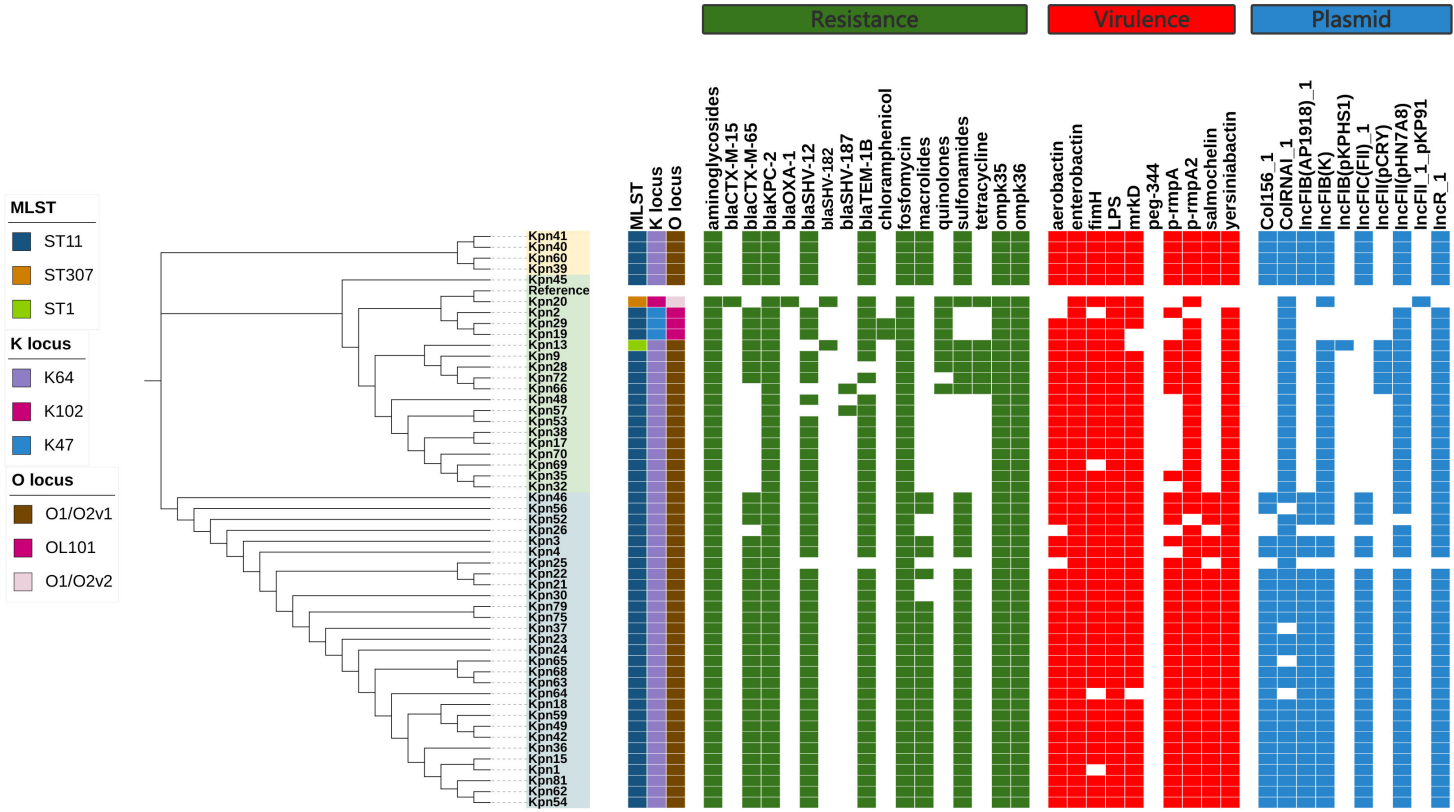
Evolutionary relationship diagram of 52 CR-hvKP strains. CR-hvKP: carbapenem-resistant hypervirulent *Klebsiella pneumonia*.

**Figure 8:**
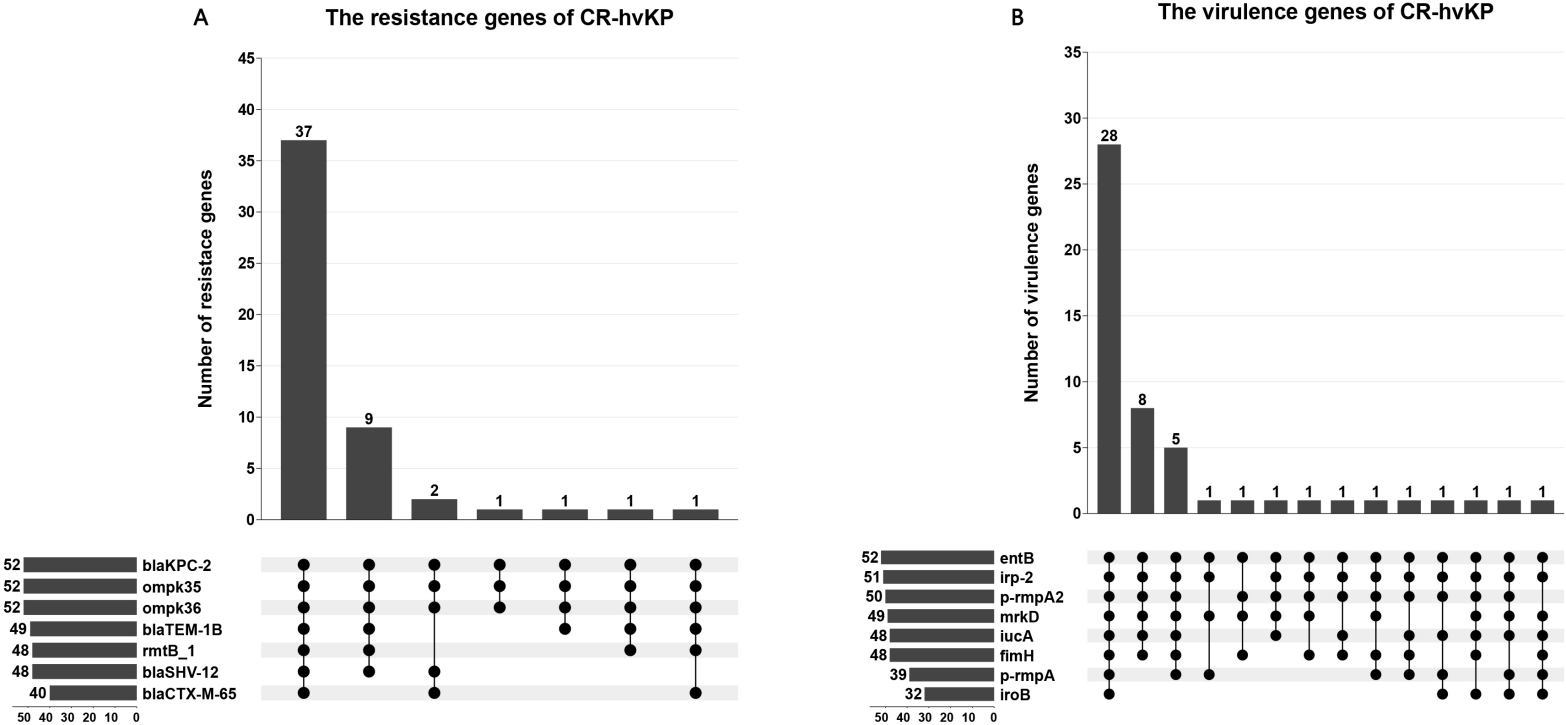
The characteristic distribution of resistance and virulence genes. **(A)** Distribution of resistance genes; **(B)** Distribution of virulence genes.

**Figure 9:**
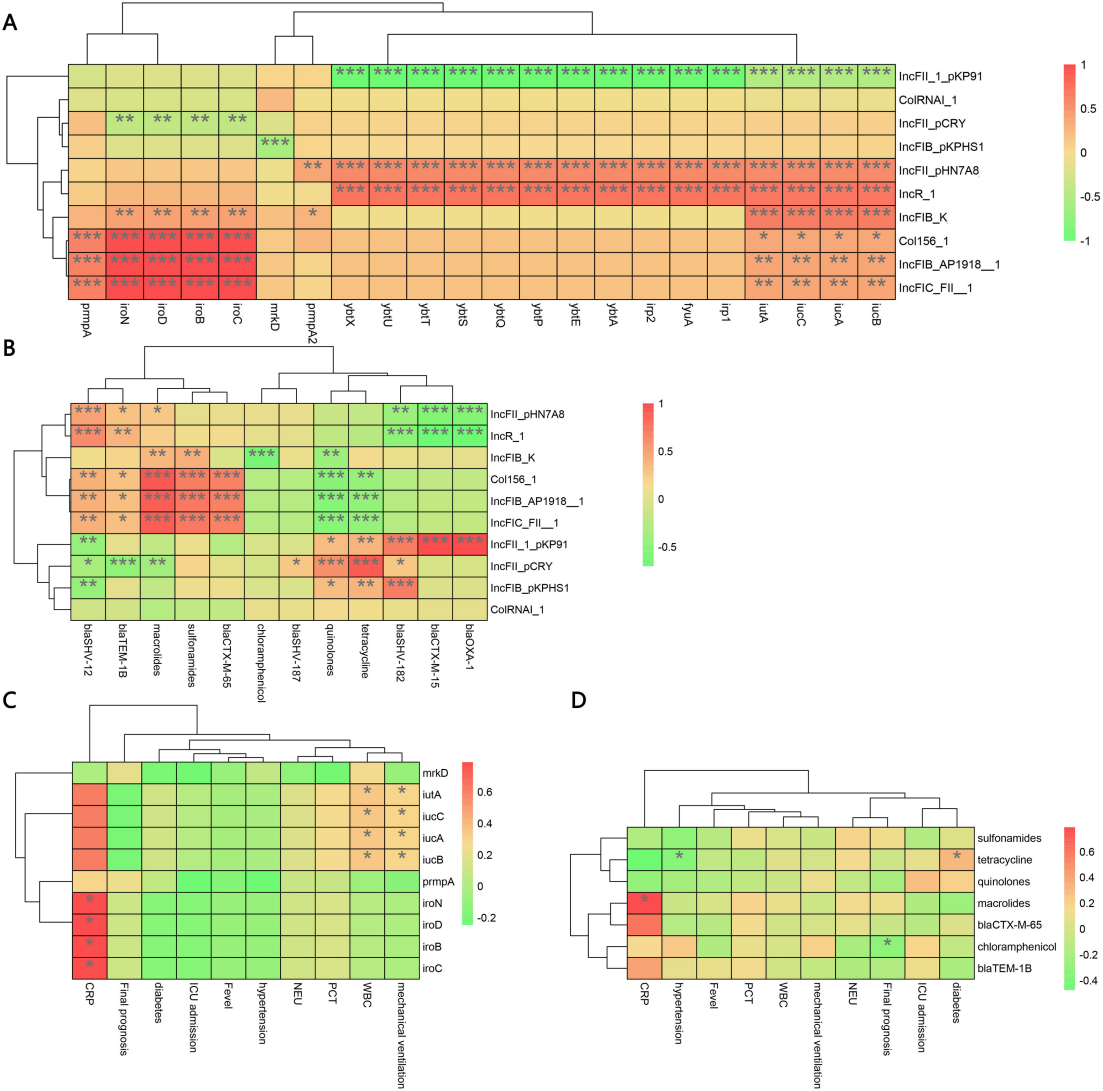
Correlations among virulence genes, resistance genes, plasmid replicons, and clinical data of the CR-hvKP strain. **(A)** Spearman correlation between virulence genes and plasmid replicon. **(B)** Spearman correlation between resistance genes and plasmid replicon. **(C)** Spearman correlation between virulence genes and clinical data. **(D)** Spearman correlation between resistance genes and clinical data. "*" represents the size of the P value, ns represents p≥0.05, *represents p<0.05, **represents p<0.01, and ***represents p<0.001. CR-hvKP: carbapenem-resistant hypervirulent Klebsiella pneumonia.

All 52 CR-hvKP strains carried the carbapenemase gene *bla*KPC-2. Carbapenem resistance genes included *bla*KPC-2 and *bla*OXA-1. Chloramphenicol resistance genes were found only in the ST11-K47-OL101 strain. The *bla*CTX-M-15 gene was detected only in ST307-K102-O1/O2v2, while SHV-182 was found in both ST307-K102-O1/O2v2 and ST1-K64-O1/O2v1. All strains carry *bla*KPC-2, *ompk*35, and *ompk*36 genes (**Figure 7**, **Figure 8A**). Carbapenem resistance genes *bla*OXA-1 and *bla*CTX-M-15 were strongly correlated with IncFII_1_pKP91, while tetracycline resistance genes were strongly associated with IncFII(pCRY) (**Figure 9B**). The resistance genes of macrolide antibiotics were positively correlated with CRP (**Figure 9D**).

Virulence gene analysis revealed that 28 CR-hvKP strains carried all tested virulence genes. The lowest detection rates were observed for *iroB* (61.54%) (**Figure 8B**). Siderophore virulence genes showed high carrying rates in all strains. Salmonella was detected in 61.54% of the strains, which was lower than that for *aerobactin* (92.31%), *yersiniabactin* (98.08%), and *enterobactin* (100%). The *yersiniabactin* gene was not detected in ST307-K102-O1/O2v2. No strains tested positive for *peg*-344 (metabolite transporter) virulence genes. Among the 52 CR-hvKP strains, 10 plasmid replicon types were identified (**Figure 7**). *Aerobactin* and *yersiniabactin* virulence genes were strongly correlated with the IncR_1 (**Figure 9A**). The virulence genes of Salmochelin were positively correlated with CRP (**Figure 9C**).

**Figure 10:**
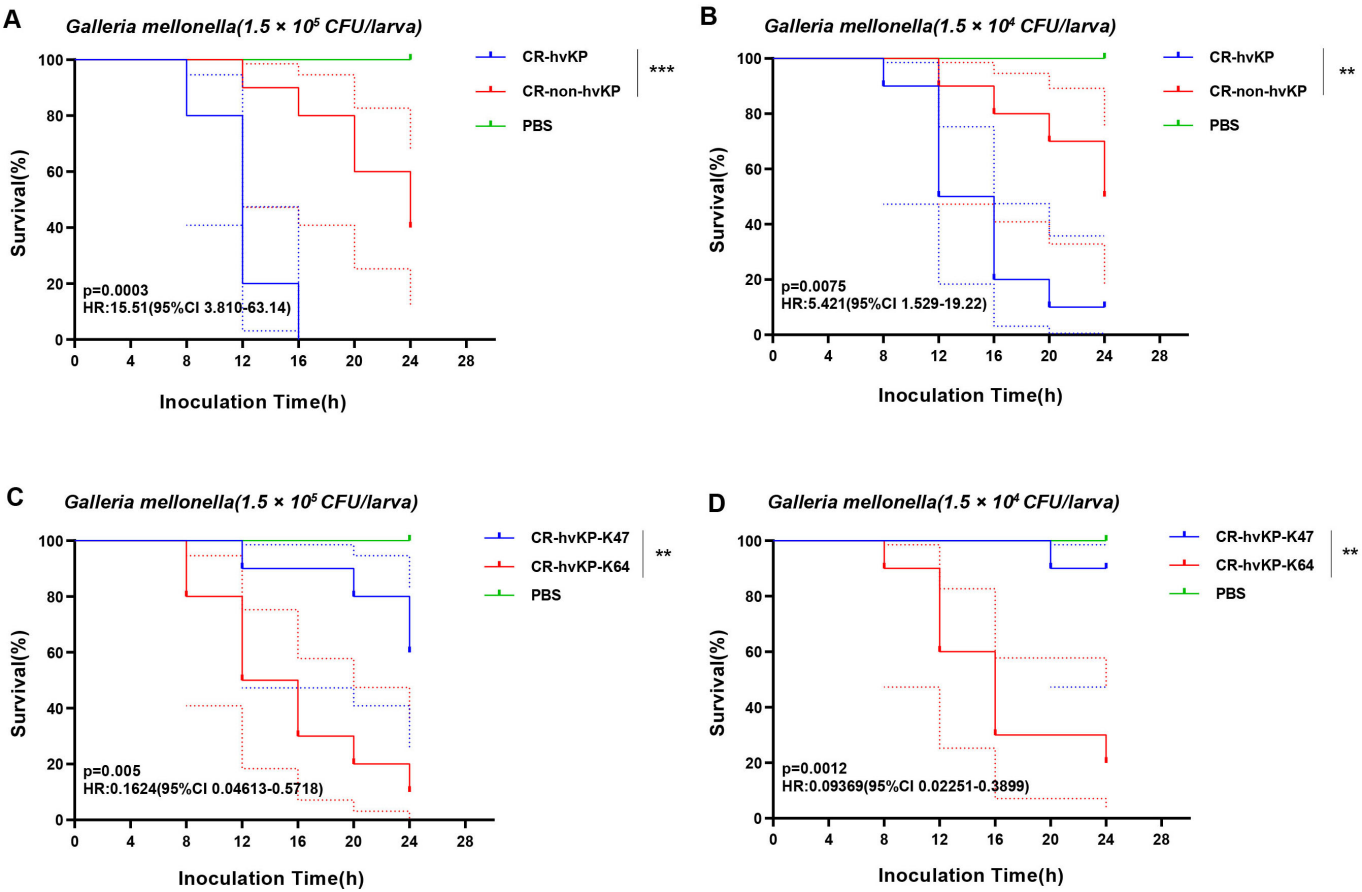
The *Galleria mellonella* infection model. **(A)** Survival rates of *Galleria mellonella* infected (1:10) with CR-hvKP and CR-non-hvKP; **(B)** Survival rates of *Galleria mellonella* infected (1:100) with CR-hvKP and CR-non-hvKP; **(C)** Survival rates of *Galleria mellonella* infected (1:10) with CR-hvKP-K47 and CR-hvKP-K64; **(D)** Survival rates of *Galleria mellonella* infected (1:100) with CR-hvKP-K47 and CR-hvKP-K64 CR-hvKP: carbapenem-resistant hypervirulent Klebsiella pneumonia CR-non-hvKP: carbapenem-resistant non-hypervirulent *Klebsiella pneumonia*. ** indicates p < 0.01, and *** indicates p < 0.001.

The experiment on the *Galleria mellonella* showed that the survival rate of CR-non-hvKP was higher than that of CR- hvKP. The survival rate of CR-hvKP-47 was higher than that of CR-hvKP-64, indicating that the virulence of CR-hvKP-64 was higher than that of CR-hvKP-47 (**Figure 10**).

### 3.5 Antibiotic resistance plasmids and mobile genetic elements

**Figure 11:**
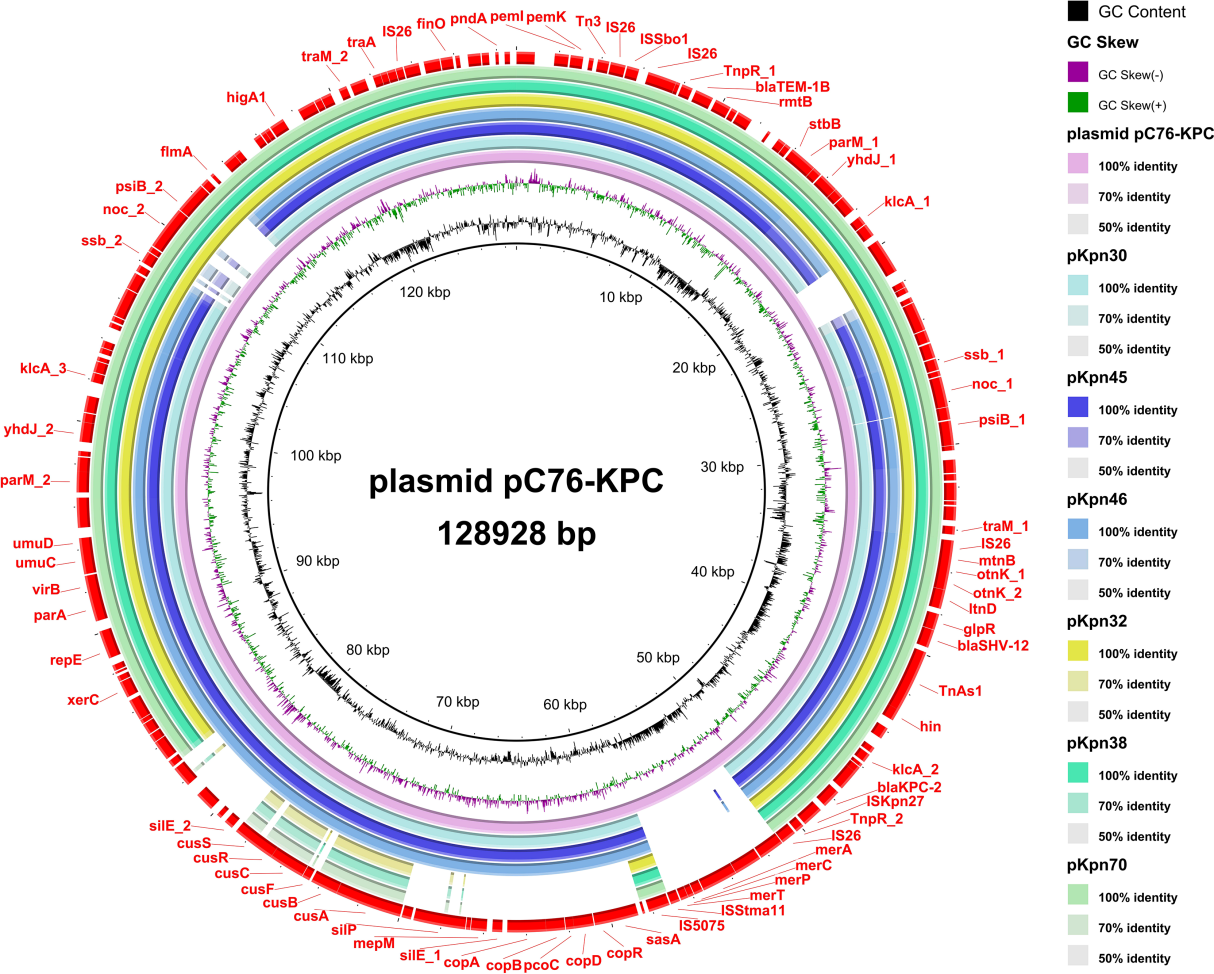
Comparative genomic analysis of antibiotic-resistant plasmids in CR-hvKP strains. From the inner circle to the outer circle, use this as follows: circle 1, GC Content; circle 2, GC Skew; circle 3, reference plasmid pC76 KPC(NZ_CP080299.1); circle 4, pKpn30; circle 5, pKpn45; circle 6, pKpn46; circle 7, pKpn32; circle 8, pKpn38; circle 9, pKpn70; and circle 10, Annotated mobile genetic elements and carbapenem resistance genes. CR-hvKP: carbapenem-resistant hypervirulent *Klebsiella pneumonia*.

**Figure 12:**
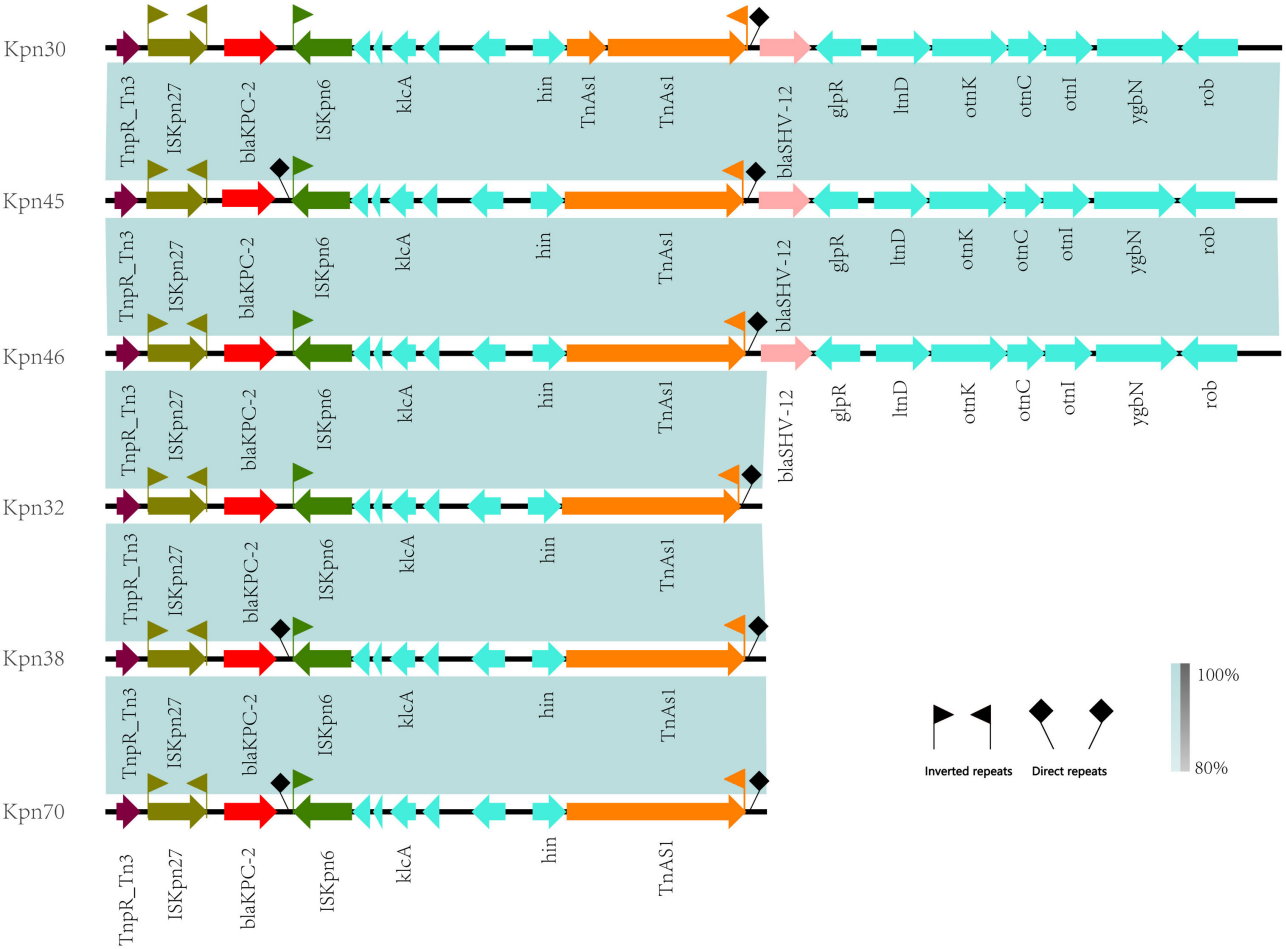
Linear alignment of gene environment in *bla*KPC-2.

Blast analysis showed that these drug-resistant plasmids were highly similar to the reference plasmid pC76 KPC (NZ_CP080299.1) with 99.37% identity and 93.25% coverage (**Figure 11**). This plasmid is located on IncFII/IncR. pKpn30, pKpn45, and pKpn46 were similar in structure and belonged to the plasmid replicons of IncFIB(AP001918)_1, IncFIC(FII)_1, and IncFII(pHN7A8)_1. The structures of pKpn32, pKpn38, and pKpn70 were similar, belonging to repB_KLEB_VIR, IncHI1B(pNDM-MAR)_1, and IncFII(pHN7A8)_1 plasmid replicons. After analysis, the *bla*KPC-2 genes of the six CR-hvKP strains were all located on plasmids and carried *bla*TEM-1B, *bla*SHV-12, and *rmtB*_1 resistance genes. Research on the genetic environment of *bla*KPC-2 has shown that its horizontal transfer is mediated by TnpR_Tn3-ISKpn27-*bla*KPC-2-ISKpn6. Various transposons and insertion sequences around the resistance genes predicted the possibility of horizontal transfer of the resistance genes. ISKpn27 contained IRL and IRR, while ISKpn6 only had IRR (**Figure 12**).

## 4 Discussion

The global prevalence of CR-hvKP is increasing (Han et al., 2022). Surveillance data from China indicate that the carbapenem resistance rate in *K. pneumoniae* has risen approximately ninefold, from 2.95% in 2005 to 25.4% in 2023, making it the second most common clinical isolate after *Escherichia coli* (https://www.chinets.com/Data/AntibioticDrugFast). A similar upward trend was observed in Europe, where resistance increased from 7.1% in 2017 to 11.7% in 2021 (https://www.ecdc.europa.eu/). CR-hvKP combines high pathogenicity with multidrug resistance, frequently resulting in poor clinical outcomes. Its capacity for rapid spread and outbreak formation poses significant challenges for infection control. Known risk factors for CR-hvKP infection include underlying chronic comorbidities, diabetes, age <65 years, and mechanical ventilation (Liang et al., 2022; Li et al., 2023).

In our hospital, the predominant sequence type was ST11, which aligns with findings from previous studies in China (Pu et al., 2023a). This stands in contrast to the epidemic ST258 clone prevalent in Europe and the United States (David et al., 2019; Ernst et al., 2020). The global epidemiology of CRKP is diverse, with ST101 reported as dominant in Turkey (Ibik et al., 2023), ST23 and ST25 linked to hypervirulence in Madagascar (Rakotondrasoa et al., 2020), and ST25 identified in Argentinean hypermucoviscous strains (Vargas et al., 2019). In Greece, ST39 carrying *bla*KPC-2 is a high-risk clone causing bloodstream infections (Tryfinopoulou et al., 2023). Geographically, the Class B carbapenemase NDM is endemic in regions including Egypt, India, Pakistan, and Serbia (Wu et al., 2019), while the Class D enzyme OXA-48 frequently cooperates with other mechanisms to enhance resistance. Notably, the ST15 clone carrying *bla*OXA-232 has spread globally from a likely origin in the United States, with significant prevalence in China (Wu et al., 2023). The emergence of CRKP strains producing multiple carbapenemases is a growing concern (Gao et al., 2020). We identified a high-risk ST307 clone lacking the siderophore genes *yersiniabactin*, *aerobactin*, and *salmochelin*. The presence of ST307 is concerning, as it has been reported that this sequence type can develop elevated MICs to CZA, leading to clinical resistance (Hernández-García et al., 2022). Furthermore, surveillance data indicate that in some regions, ST307 has replaced established high-risk clones such as ST512 and ST258 (Peirano et al., 2020). These findings from other settings necessitate heightened vigilance and monitoring for the emergence of this clone in our region.

*K. pneumoniae* expresses over 79 K-capsule types. In China, K64 (50.4%) and K47 (25.9%) are the most common among CRKP, with K64 increasing in eastern and central China while K47 declines, now primarily found in the north and northeast (Hu et al., 2024). Capsule-deficient strains exhibit impaired transmission and phagocytosis resistance but may display enhanced antibiotic resistance, causing persistent urinary tract infections. Conversely, hypercapsular strains show increased resistance to phagocytosis, dissemination, and mortality (Hu et al., 2024; Ernst et al., 2020). The evolving serotype prevalence underscores the urgent need for CRKP vaccines. Our finding that CR-hvKP-K64 is more virulent than CR-hvKP-K47 is consistent with that of the report by Jia et al (Jia et al., 2021).

Avibactam is a non-β-lactam β-lactamase inhibitor active against serine β-lactamases but not metallo-β-lactamases. Due to its efficacy and safety against serine carbapenemases, CZA has become a first-line treatment for CRKP (Tumbarello et al., 2021). However, CZA resistance is emerging, driven by *bla*KPC mutations, including *bla*KPC-135 and *bla*KPC-112, frequently selected by antibiotic pressure (Shi et al., 2024; Shen et al., 2022). The spread of *bla*KPC involves multiple mechanisms, including mobile genetic elements, plasmid transfer, and clonal spread. In our study, CZA resistance was primarily mediated by a *bla*KPC-2-bearing plasmid, pC76 KPC (NZ_CP080299.1), a non-classical type first cataloged in the RefSeq database in the United States (Tatusova et al., 2014). The *bla*KPC gene is typically mobilized by elements, including the Tn4401 transposon (common in the USA) and the NTEKPC-I/II elements (common in China and Brazil) (Naas et al., 2012). Our analysis of the *bla*KPC-2 genetic context revealed flanking insertion sequences ISKpn27 and ISKpn6. The presence of incomplete reverse repeats adjacent to ISKpn27 may facilitate gene mobilization. Different *bla*KPC-2 subtypes are associated with distinct mobile elements and plasmids, highlighting the complexity of their dissemination.

Beyond CZA resistance, we observed that polymyxin resistance rates were lower than those for tigecycline. However, hypervirulent ST11-K64 can rapidly develop resistance during tigecycline or polymyxin therapy (Jin et al., 2021). The emergence of the *tmexCD-toprJ* gene complex further threatens tigecycline efficacy (Yao et al., 2024). Concurrently, increasing reports of polymyxin-resistant, hypervirulent *K. pneumoniae* pose a major public health challenge (Liu et al., 2022). Given these multifaceted resistance threats, novel therapeutic approaches, including non-ribosomal tobramycin-cyclohexane conjugates, could enhance the efficacy of β-lactam/β-lactamase inhibitor combinations (Idowu et al., 2019), offering a promising direction for future research.

This study had some limitations that should be considered. First, the single-center design may mean that the observed clonal composition is specific to our local epidemiology and antibiotic practices. Second, while providing detailed insights, the sample size may not capture the full diversity of CR-hvKP in the region. These factors could limit the generalizability of our results. To overcome these constraints, future research will focus on employing long-read sequencing for complete genomic analysis and expanding into a multi-center study to verify the national relevance of our findings.

## 5 Conclusion

In conclusion, our study underscores the pivotal role of the ST11-K64 clone in converging CR-hvKP in China, linked clinically to prolonged antibiotic exposure and malignancies. We delineated a novel plasmid-borne genetic context (TnpR_Tn3-ISKpn27-*bla*KPC-2-ISKpn6) responsible for high-level CZA resistance. These findings highlight an urgent need for enhanced surveillance and stringent infection control to contain this public health threat. Future efforts should focus on developing rapid diagnostics for these resistance mechanisms and exploring novel combination therapies.

## Data Availability

The datasets presented in this study can be found in online repositories. The names of the repository/repositories and accession number(s) can be found below: https://www.ncbi.nlm.nih.gov/genbank/, BioProject number:PRJNA1200658.
